# Assessment of Trihalomethane Formation in Chlorinated Raw Waters with Differential UV Spectroscopy Approach

**DOI:** 10.1155/2013/890854

**Published:** 2013-12-02

**Authors:** Kadir Özdemir, İsmail Toröz, Vedat Uyak

**Affiliations:** ^1^Department of Environmental Engineering, Bulent Ecevit University, Incivez, 67100 Zonguldak, Turkey; ^2^Department of Environmental Engineering, Istanbul Technical University, Maslak, 34469 Istanbul, Turkey; ^3^Department of Environmental Engineering, College of Engineering, Pamukkale University, Kinikli, 20020 Denizli, Turkey

## Abstract

In this study, the changes in UV absorbance of water samples were characterized using defined differential UV spectroscopy (DUV), a novel spectroscopic technique. Chlorination experiments were conducted with water samples from Terkos Lake (TL) and Büyükçekmece Lake (BL) (Istanbul, Turkey). The maximum loss of UV absorbance for chlorinated TL and BL raw water samples was observed at a wavelength of 272 nm. Interestingly, differential absorbance at 272 nm (ΔUV_272_) was shown to be a good indicator of UV absorbing chromophores and the formation of trihalomethanes (THMs) resulting from chlorination. Furthermore, differential spectra of chlorinated TL waters were similar for given chlorination conditions, peaking at 272 nm. The correlations between THMs and ΔUV_272_ were quantified by linear equations with *R* ^2^ values >0.96. The concentration of THMs formed when natural organic matter is chlorinated increases with increasing time and pH levels. Among all THMs, CHCl_3_ was the dominant species forming as a result of the chlorination of TL and BL raw water samples. The highest chloroform (CHCl_3_), dichlorobromomethane (CHCl_2_Br), and dibromochloromethane (CHBr_2_Cl) concentration were released per unit loss of absorbance at 272 nm at pH 9 with a maximum reaction time of 168 hours and Cl_2_/dissolved organic carbon ratio of 3.2.

## 1. Introduction

Disinfection of surface water supplies containing natural organic matter (NOM) with chlorine leads to formation of chlorinated brominated, and in much smaller levels, iodinated by-products defined as disinfection by-products (DBPs) [1–3]. Trihalomethanes (THMs) and halo acetic acids (HAAs) are the main groups of DBPs commonly found in drinking waters [4–7]. Such hazardous compounds have been shown to be related to the occurrence of cancer, growth retardation, spontaneous abortion, and congenital cardiac effects [8–13]. Therefore, strict regulations for water quality have been recently imposed in some European countries [14]. These regulations should ensure the safety of drinking water through the elimination (or reduction to a minimum concentration) of the hazardous substances in water. The maximum contaminant level of THMs was set to 80 *μ*g/L by United States Environmental Protection Agency (USEPA) [15]. Whereas the European Union (EC) has set the THMs limit to 100 *μ*g/L [16]. The THMs limit in Turkey is also 100 *μ*g/L [17].

The relationships among chlorination conditionals such as pH, temperature, reaction time, bromide concentration, chlorine dosage and NOM concentration, and the formation of DBPs are highly nonlinear and complex [18]. Developing formal kinetic or statistical models for DBP formation currently requires substantial cost and effort associated with analysis of DBPs, thus restricting the amount of data that can be obtained from any single laboratory or field study of the chlorination reactions and limiting the availability of information that may be useful in formulating or testing models of the reaction sequence [19]. Several researches have attempted to correlate water quality parameters to DBP formation in an effort to find a useful surrogate parameter to predict DBP formation or to better understand the chemical nature of DBP formation processes [20–22]. The use of surrogate parameters to monitor formation of chlorinated by-products can be used as an alternative to mechanistic or statistical models for estimating DBPs formation. Many surrogate parameters that have been most widely used to estimate DBP formation potential (DBPFP) include ultraviolet absorbance (UV), specific UV absorbance (SUVA), which is UV absorbance divided by dissolved organic carbon (DOC) concentration and DOC. It was reported that the correlation between UV absorbance at 254 nm (UV_254_) wavelength and the THM formation potential (THMFP) was strong [23].

Since SUVA is strongly correlated with the aromaticity and reactivity of NOM, it has been used extensively as a conventional parameter [24, 25] and can therefore be used to estimate the concentration of NOM moieties in a water sample.

It has been reported that simple and reliable relationships existed between change in UV absorbance of NOM and formation of DBPs during the chlorination processes [21, 26–28]. As the aromatic functional groups are thought to be both dominant chromospheres in NOM and the dominant sites of attack by chlorine on NOM molecules, UV_254_ has frequently been proposed to predict the concentration of DBP precursors. Although the use of UV spectroscopy to estimate DBP formation is problematic, a technique known as differential UV spectroscopy (DUV) has been developed [29]. ΔUV has been shown to be an effective spectrophotometric method for monitoring the amount of DBPs formed by chlorination of NOM. This approach focuses on the change in UV absorbance caused by the chlorination reaction, rather than the overall UV spectrum of water. The differential UV spectrum of chlorinated NOM is defined as shown in

(1) $$\begin{array}{c} \Delta {\text{UV}}_{\lambda }={\text{UV}}_{\lambda }^{\text{chlorinated}}-{\text{UV}}_{\lambda }^{\text{inital}}, \end{array}$$

where UV_*λ*_^inital^ is the UV absorbance at wavelength *λ* prior to chlorination, UV_*λ*_^chlorinated^ is the UV absorbance at wavelength *λ* after chlorination, and ΔUV_*λ*_ is the differential UV absorbance at wavelength *λ*.

As the chlorination reaction with NOM occurs primarily at sites that absorb UV light, DUV could provide a sensitive and highly specific probe for chlorination reactions. Moreover, the magnitude of decrease in UV absorbance at 272 nm (ΔUV_272_) was found to be an excellent indicator of total organic halogen formation resulting from chlorination, independent of chlorine to DOC ratio, bromide concentration, pH, reaction time, and NOM source [21, 30, 31].

In this study, we investigated the applicability of differential absorbance to quantify the reactivity of NOM from raw waters.

## 2. Materials and Methods

### 2.1. Sample Collection

During this study, water samples were taken from Terkos and Büyükçekmece Lakes in Istanbul, Turkey. Samples were collected during the summer period (June, July, and August) in 2010. Terkos Lake (TL) and Büyükçekmece Lake (BL) are the main surface water sources of Istanbul, providing nearly 1 million m^3^ raw water to the drinking water treatment plants of Kağıthane and Büyükçekmece. The characteristics of raw water quality parameters are presented in Table 1. Raw water samples were collected as grab samples and stored in a refrigerator at 4°C to retard biological activity.

### 2.2. Chlorination Procedure

Chlorination of raw water samples was conducted in accordance with Standard Methods 5710 B [32]. Before chlorination, sample pH values were adjusted to pH 5, 7, and 9 by addition of HCl or NaOH solution. The chlorinated samples were placed into 125 mL amber glass bottles with polypropylene screw caps and TFE-faced septa. Raw water samples were chlorinated to Cl_2_/DOC ratios of 0.8, 1.6, and 3.2 before incubation in the dark for either 1, 4, 24, 48, 96, or 168 hours. After the reaction periods, chlorine residual concentrations were determined with DPD ferrous titrimetric method according to Standard Methods 4500 Cl-F [32]. Sodium sulfite solution was used as a quenching agent for all chlorinated samples prior to UV spectrophotometric and THM analyses.

### 2.3. Analytical Procedure

THM measurements were performed by liquid-liquid extraction (LLE) with n-pentane. For THMs, a total of six THM calibration standards were prepared using certificated commercial mix solutions (Accu Standard, Inc., purity >99%). Samples were analyzed by gas chromatography (GC) equipped with a microelectron capture detector (*μ*ECD) for THM analyses. A capillary column of (DB-1 J&W Science) 30 m × 0.32 mm and 1.0 *μ*m film thickness was used. Samples were injected in split/splitless mode with helium as a carrier gas and nitrogen gas as a make-up gas. The minimum quantification limits for THM species ranged between 0.5 and 1 *μ*g/L. DOC concentrations were measured on a Schimadzu 5000 total carbon analyzer equipped with an AS auto sampler according to method 5310 B in Standard Methods [32]. The instrument provided reliable, accurate, and reproducible data with a minimum detection limit of 2 *μ*g/L. The UV absorbance readings between wavelengths of 250 and 410 nm were determined using a Shimadzu 1608 UV/VIS spectrophotometer.

## 3. Result and Discussion

### 3.1. Differential UV Spectra of Chlorinated NOM

**Figure 1:**
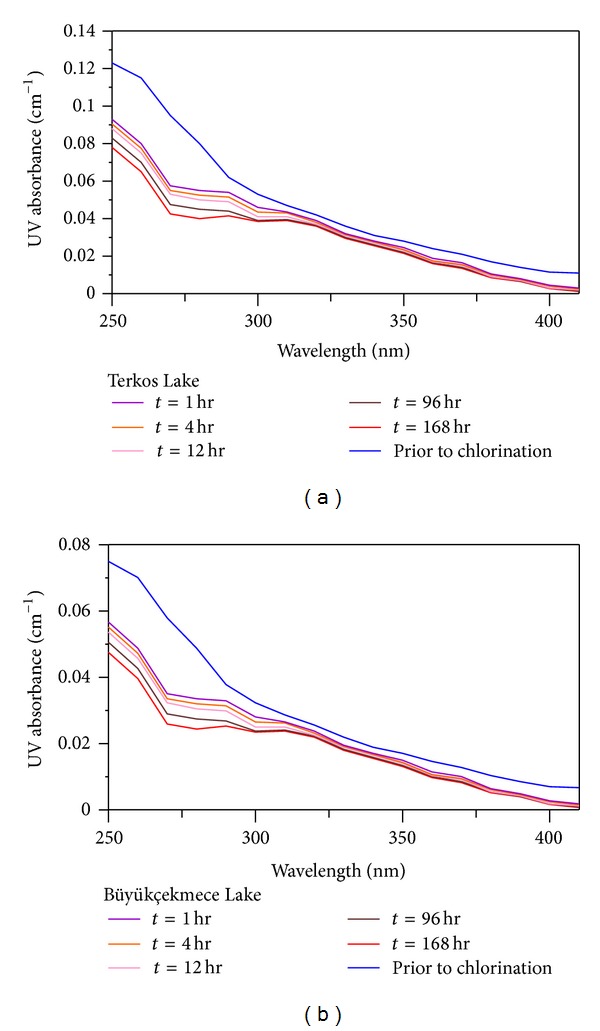
Chlorinated UV absorbance spectra at varying reaction times and at pH 7 for (a) TL water samples and (b) BL water samples.

The changes of UV absorbance spectra for TL and BL water samples, including NOM before and after chlorination, are shown in Figure 1. The UV absorbance values of TL and BL water samples at given wavelengths (250–400 nm) are significantly decreased for different reaction times (1, 4, 12, 96, and 168 hours) after chlorination. For instance, the UV absorbance of TL raw water samples at a wavelength of 272 nm was reduced from 0.095 to 0.06 cm^−1^ by 1 h chlorination. Whereas the UV absorbance of BL raw water samples at the same wavelength decreased from 0.064 to 0.032 cm^−1^ by 1 h after chlorination.

The similar trend with corresponding to UV spectra of chlorinated TL and BL raw water samples was determined at the range of 250–400 nm for the desired reaction times. The maximum loss of UV absorbance in TL and BL water samples was recorded at 400 nm and 168 h reaction time, reaching 0.0012 and 0.001 cm^−1^, respectively. The ratio of decreasing UV absorbance of chlorinated water samples containing NOM followed consistent pattern as a function of wavelength and at a given reaction time.

**Figure 2:**
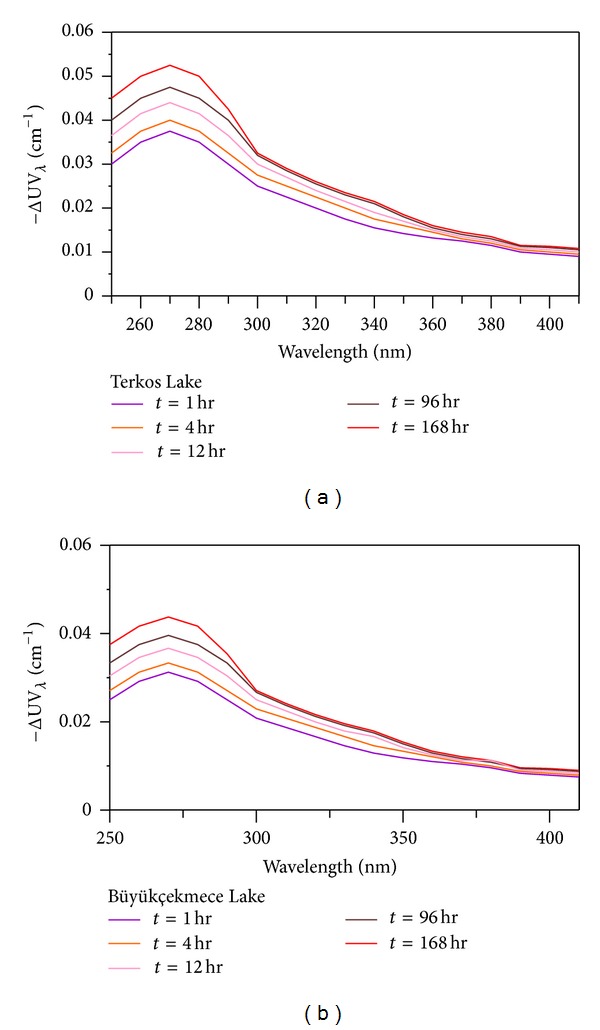
Differential spectra at varying reaction times and at pH 7 for (a) TL water samples and (b) BL water samples.

Changes in the UV spectrum of chlorinated NOM-containing TL and BL raw water samples are presented with differential absorbance spectrum between 250 and 400 nm (Figure 2).

The measurement of differential UV spectrum of TL and BL raw water samples was characterized using (1). These findings demonstrate several shared properties of the differential spectra. The sign of differential absorbance was always negative at 250–400 nm wavelengths because the UV absorbance of NOM surface water supplies decreased with chlorination. The differential spectra of chlorinated TL and BL raw water samples consistently peak at 272 nm. In fact, 272 nm was the maximum differential absorbance for all chlorinated TL and BL raw water samples at all reaction times. The TL and BL differential absorbance values of water samples at 272 nm at a reaction time of 4 h were 0.04 and 0.0285 cm^−1^, respectively. The highest ΔUV_272_ values were observed at a reaction time of 168 h (0.0525 and 0.04 cm^−1^ for TL and BL samples, resp.), demonstrating that the magnitude of the differential spectra developed with increasing chlorination reaction time, and is consistent with the results of other studies [28, 29, 33].

We also found that the differential spectrum of chlorinated TL and BL water including NOM was related to other independence parameters of water quality and chlorination conditions. Furthermore, these spectra exhibited a peak at 272 nm, suggesting that differential absorbance is an effective spectrophotometric parameter providing insight into the reactivity of NOM molecules with regard to the formation of DBPs such as THMs. An observation was also made in previous publications [21, 31, 34, 35].

### 3.2. Relationship between ΔUV_272_ and THM Concentrations

**Figure 3:**
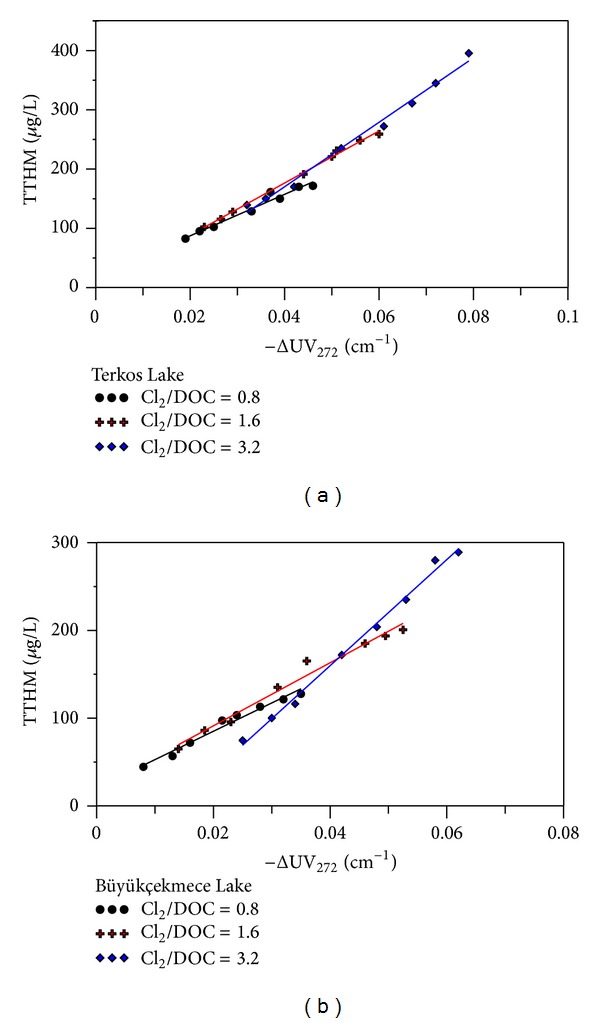
Correlations between ΔUV_272_ values and TTHM concentrations at different Cl_2_ to DOC ratios, pH 7, and reaction times from 1 h to seven days for (a) TL water and (b) BL water samples.

A series of experiments were conducted upon TL and BL water samples to examine THM formation at three Cl_2_/DOC ratios (0.8, 1.6, and 3.2), for the chlorination pH (pH = 7) and at reaction times from 1 h to 168 h. We identified strong correlations between total THM (TTHM) concentrations and ΔUV_272_ values obtained by the chlorination of TL raw waters (Figure 3).

These correlations were modeled by linear regression analysis with *R* ^2^ values >0.98 given by the following:

TL waters

(2) $$\begin{array}{c} {\text{TTHM}}^{}\left(\mu \text{g}/\text{L}\right)=4952.2^{}\;\Delta {\text{UV}}_{272}-25.12. \end{array}$$

BL raw waters

(3) $$\begin{array}{c} {\text{TTHM}}^{}\left(\mu \text{g}/\text{L}\right)=4339.5^{}\;\Delta {\text{UV}}_{272}-3.08. \end{array}$$

The highest TTHM content and ΔUV_272_ value (395.62 *μ*g/L and 0.079 cm^−1^, resp.) were obtained for the chlorinated TL raw water samples with the SUVA level of 3.04 L/mg·m, at the maximum Cl_2_/DOC ratio (3.2) and reaction time (168 h). The relationship between hydrophobic SUVA and the formation of TTHM is dependent on the activated aromatic structures which are the major components in NOM. In high SUVA water (e.g., TL raw water), high chlorine reactivity resulted in the formation of more THMs than in low SUVA level waters (e.g., BL raw water that has hydrophilic organic fractions).

### 3.3. The Effects of pH, Reaction Time, and Chlorine Dose on THMs Formation

**Figure 4:**
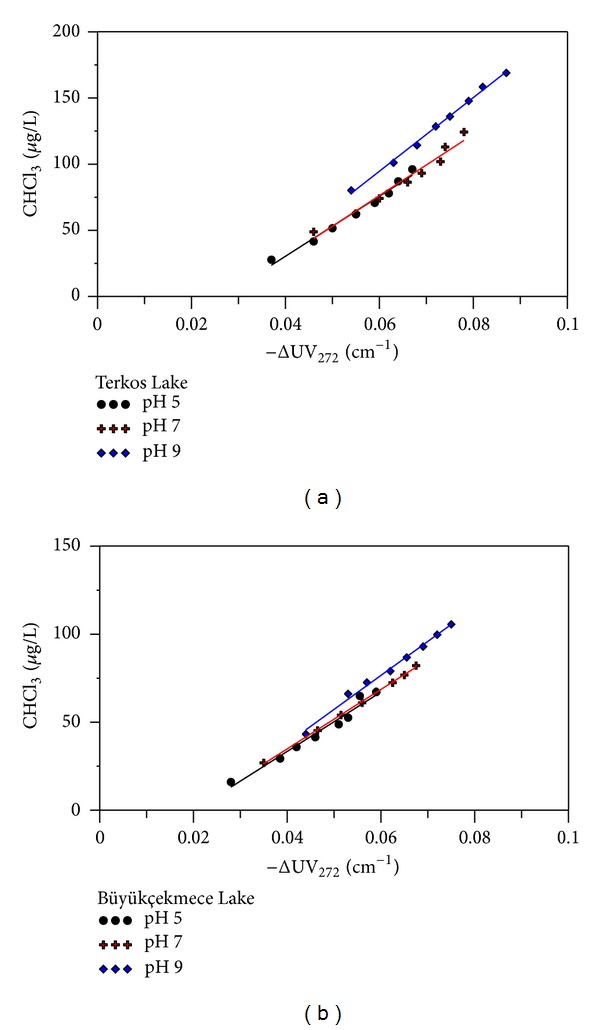
The relationship between CHCl_3_ and ΔUV_272_ at different pH (pH 5, pH7, and pH 9) and Cl_2_ to DOC ratios from 0.8 to 3.2 (a) in chlorinated TL water and (b) for in chlorinated BL water.

Solution pH has a significant effect on the speciation and amount of THM forming as a result of chlorination. We found that higher pH resulted in increased THM concentrations for the two surface water sources studied. CHCl_3_, dichlorobromomethane (CHCl_2_Br), and dibromochloromethane (CHBr_2_Cl) are common THM compounds in chlorinated TL and BL raw waters. The concentrations of CHCl_3_ generated in chlorinated TL and BL raw waters, as estimated by ΔUV_272_ and at pHs 5, 7, and 9 are shown in Figure 4.

**Figure 5:**
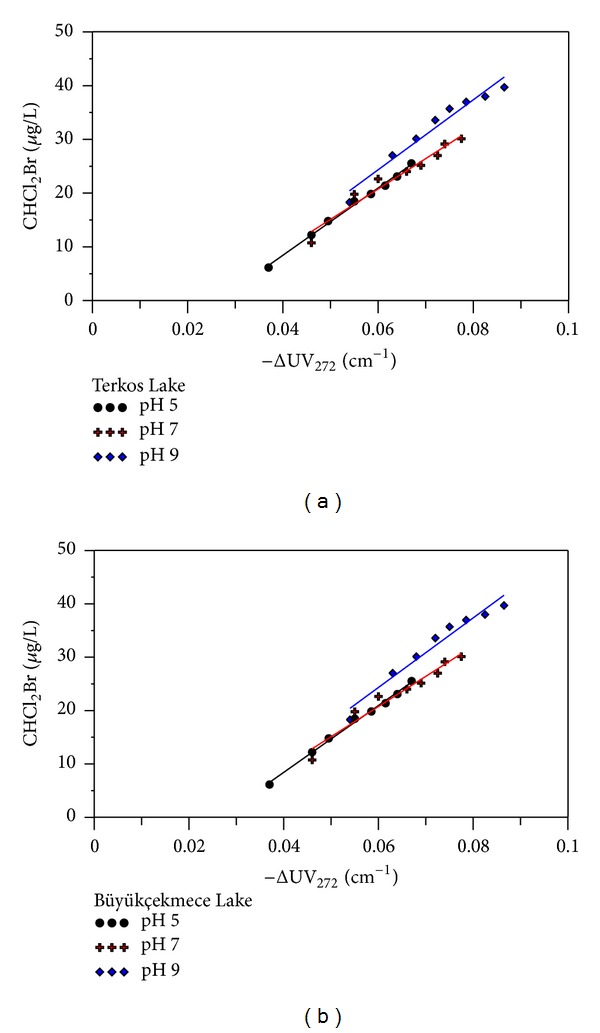
The relationship between CHCl_2_Br and ΔUV_272_ at different pH (pH 5, pH7, and pH 9) and Cl_2_ to DOC ratios from 0.8 to 3.2 (a) for in chlorinated TL water and (b) for in chlorinated BL water.

**Figure 6:**
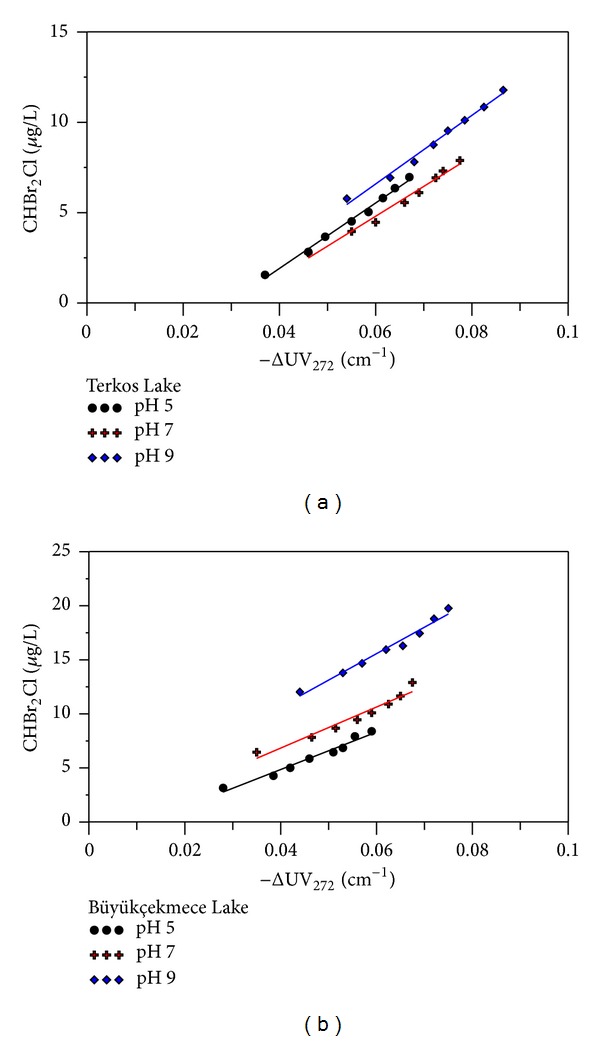
The relationship between CHClBr_2_ and ΔUV_272_ at different pH and Cl_2_ to DOC ratios (a) in chlorinated TL water and (b) in chlorinated BL water.

The relationship between CHCl_3_ concentrations and ΔUV_272_ could be well fitted by a straight line (*R* ^2^ > 0.98). As the pH increased for each raw water source, more CHCl_3_ was formed per unit of UV absorbance destroyed. Furthermore, the highest CHCl_3_ concentration and ΔUV_272_ absorbance value (222.55 *μ*g/L and 0.0865 cm^−1^, resp.) were measured at the highest pH level tested (TL water, pH 9, Cl_2_/DOC 3.2, and 168 h reaction time). Whereas the lowest CHCl_3_ concentration and ΔUV_272_ value (67.16 *μ*g/L and 0.059 cm^−1^, resp.) were measured at the most acidic pH (BL water, pH 5, Cl_2_/DOC 3.2, 168 h reaction time). This can be explained using the mechanism of DBPs formation as described by Reckhow and Singer [36]. According to this mechanism, base-catalyzed hydrolysis prevails under alkaline conditions, resulting in more CHCl_3_ at pH 9, relative to acidic conditions (pH 5). Although CHCl_3_ formation to ΔUV_272_ regression lines did not pass through the origin for all pHs studied, this can be explained by some initial reactions between NOM and Cl_2_. These reactions demolish the aromaticity of activated functional groups and produce chlorinated intermediates before CHCl_3_ formation [29, 37, 38]. CHCl_2_Br and CHBr_2_Cl were the other major THMs identified in chlorinated TL and BL raw waters. The concentrations of CHCl_2_Br and CHBr_2_Cl were also found to increase with increasing pH levels with the highest CHCl_2_Br and CHBr_2_Cl concentrations (39.72 and 19.77 *μ*g/L) found at pH 9 at 168 h reaction time and Cl_2_/DOC ratio of 3.2. Despite relatively low concentrations of CHCl_2_Br and CHBr_2_Cl in the chlorinated water samples, there was a strong linear correlation between CHCl_2_Br and CHBr_2_Cl and ΔUV_272_ (Figures 5 and 6).

## 4. Conclusion

The chlorination of TL and BL water samples including NOM resulted in decreasing UV absorbance at all wavelengths due to the destruction of UV-absorbing chromospheres and produced characteristic UV spectra. The shape of these differential UV spectra for the chlorinated TL and BL water samples was similar for the given chlorination conditions. In particular, the maximum loss of UV absorbance for the chlorinated raw water samples was observed at 272 nm. This study also examined the relationships between ΔUV_272_ and the formation of THMs in chlorinated waters from TL and BL water sources for Istanbul, Turkey.

The correlation between ΔUV_272_ and TTHM was quantified by linear regression analysis, showing *R* ^2^ values >0.96. The highest TTHM and THM contents of raw water samples were observed at pH 9 (the highest value tested).

Among the THM species formed by chlorination of the TL and BL raw water samples, CHCl_3_ was predominant. The highest concentrations of THM compounds, as measured by unit loss of absorbance at 272 nm, were produced at the highest chlorine dosage, maximum reaction time, and pH 9.

These results demonstrate that ΔUV_272_ is an alternative approach for the continuous and instantaneous monitoring of THM formation under a wide range of chlorination conditions and water qualities.

## Figures and Tables

**Table 1:** Raw water quality parameters.

Parameter	Unit	Terkos Lake	Büyükçekmece Lake
		Average value	
pH	—	7.97 ± 0.16	8.19 ± 0.14
Turbidity	NTU	3.34 ± 0.46	3.24 ± 0.27
Total Hardness	mg CaCO_3_/L	116.3 ± 6.7	166.4 ± 10.3
Alkalinity	mg CaCO_3_/L	103.1 ± 7.53	114 ± 7.7
Temperature	°C	17.2 ± 2.3	17.1 ± 2.1
DOC	mg/L	4.78 ± 0.3	4.71 ± 0.45
UV254	cm^−1^	0.13 ± 0.01	0.095 ± 0.008
Br	*μ*g/L	90 ± 20	180 ± 20
DBPFP	*μ*g/L	278 ± 30.2	230 ± 24.4
Conductivity	*μ*S/cm	305 ± 13.5	470 ± 16
SUVA	L/mg·m	3.04 ± 0.14	2.02 ± 0.23
